# Impact of Bio-Carrier Immobilized with Marine Bacteria on Self-Healing Performance of Cement-Based Materials

**DOI:** 10.3390/ma13184164

**Published:** 2020-09-19

**Authors:** Hayeon Kim, Hyeongmin Son, Joonho Seo, H. K. Lee

**Affiliations:** Department of Civil and Environmental Engineering, Korea Advanced Institute of Science and Technology, 291 Daehak-ro, Yuseong-gu, Daejeon 34141, Korea; gkdus305@kaist.ac.kr (H.K.); nemilhm@kaist.ac.kr (H.S.); junhoo11@kaist.ac.kr (J.S.)

**Keywords:** self-healing efficiency, bio-carrier, ureolytic bacteria, immobilization, microbial viability

## Abstract

The present study evaluated the self-healing efficiency and mechanical properties of mortar specimens incorporating a bio-carrier as a self-healing agent. The bio-carrier was produced by immobilizing ureolytic bacteria isolated from seawater in bottom ash, followed by surface coating with cement powder to prevent loss of nutrients during the mixing process. Five types of specimens were prepared with two methods of incorporating bacteria, and were water cured for 28 days. To investigate the healing ratio, the specimens with predefined cracks were treated by applying a wet–dry cycle in three different conditions, i.e., seawater, tap water, and air for 28 days. In addition, a compression test and a mercury intrusion porosimetry analysis of the specimens were performed to evaluate their physico-mechanical properties. The obtained results showed that the specimen incorporating the bio-carrier had higher compressive strength than the specimen incorporating vegetative cells. Furthermore, the highest healing ratio was observed in specimens incorporating the bio-carrier. This phenomenon could be ascribed by the enhanced bacterial viability by the bio-carrier.

## 1. Introduction

Securing the long-term durability of marine concrete structures, which are difficult to maintain, is an important issue. One of the ultimate factors affecting the durability of concrete structures is the occurrence of micro-cracks [1,2,3,4,5]. In particular, the infiltration of chloride ions into the micro-cracks could destroy the passive film of rebars embedded in reinforced concrete structures due to electrochemical reactions, thereby causing the corrosion of rebars [6,7,8]. In addition, sulfate ions could change the pore size distribution of concrete by forming ettringite, meaning that the various ions (i.e., CO_3_^2−^, SO_4_^2−^, Cl^−^, Ca^2+^, Mg^2+^, Na^+^, etc.) present in seawater could induce the change in hydration products or mineral phases in the concrete [7,9]. Crack-closure work, therefore, is fundamental to protect the concrete from deterioration factors and to improve the long-term durability. In general, there is an autogenous healing system of concrete itself; however, its crack healing efficiency depends on environmental factors and the crack width that can be filled is restricted [3,10,11].

As a new concept of self-healing technology, the microbial metabolism-based crack healing mechanism has garnered attention. Bacteria incorporated in concrete can be activated when water, air, and nutrients are introduced through micro-cracks, and then can generate carbonate ions using bacterial inherent enzyme-based metabolism [12,13,14]. The carbonate ions can combine with metal ions present in seawater or in cement matrix, which form the carbonate minerals and, finally, fill the cracks [15,16,17]. Some researchers have reported that biogenic carbonate minerals which have filled in cracks bond strongly with cement grains, resulting in a high strength recovery rate [18,19]. Therefore, bacteria can be anticipated as a sustainable crack healing agent, since they could continuously perform the metabolism and could be mass-cultured [20], suggesting that the self-healing system using bacterial metabolism is eco-friendly and prospective technology for long-term durability.

Meanwhile, bacteria incorporated into cementitious materials could be vulnerable to extreme environments formed as the hydration of cement proceeds, including such environmental factors as high temperature, pressure, and alkalinity [18,21]. Accordingly, the method of incorporating bacteria is a critical aspect for the long-term self-healing performance in microbial metabolism-based self-healing concrete. Many researchers have introduced various bio-carriers for protecting the microbial cells from the harsh environment in concrete [19,22,23,24,25,26]. Xu and Wang (2018) [27] prepared encapsulation materials by mixing calcium sulphoaluminate cement, which has a low alkalinity, with silica fume and a spore solution of *Sporosarcina pasteurii*. They confirmed that the crack widths of 0.417 mm were completely healed after 28 days, and the strength of the specimens with the bio-carrier was increased by 130% [27]. Xu et al. (2019) [28] used rubber particles immobilized with bacterial paste as a self-healing agent, and reported higher slump, tensile strength, and anti-cracking potential in specimens that incorporated the bio-carrier.

In the present study, a novel bio-carrier immobilized with bacteria was proposed to secure the sustainable self-healing efficiency. Furthermore, ureolytic bacteria to be immobilized were isolated from seawater, considering the geographic location of marine concrete structures, and their metabolism properties were identified. Specimens were prepared by various mix proportions according to the method of incorporating bacteria and whether the normal carrier or the bio-carrier was incorporated, followed by 28 days of water curing. Compressive strength and mercury intrusion porosimetry (MIP) tests were performed, and then the healing ratio through a wet–dry cycle under three different environments (seawater, tap water, and air) was evaluated via microscopic observation.

## 2. Experimental Procedure

### 2.1. Identification of Ureolytic Bacteria Isolated in Seawater

To isolate the ureolytic bacteria in a marine environment, sampling from the West Sea (36°14′47.9″N 126°32′13.3″E) and East Sea (36°02′09.3″N 129°22′56.0″E) in South Korea was conducted. The ureolytic bacteria were identified using a urease test medium to detect the urease enzyme production by a color transition. The urease test medium consisted of 2.9% urea agar base (Becton Dickinson, Franklin Lakes, NJ, USA), 1.8% agar, and NaCl added to reach 3% salinity of the final concentration. The urea agar base was sterilized by the 0.22-μm membrane filter method to prevent the urea degradation by the autoclaving process. The pure single colonies were selected through a repetitive streaking plate method after 5 days of incubation.

In order to identify the selected ureolytic bacteria, sequencing analysis of 16s rRNA was conducted. A sampling process for DNA extraction was carried out when the isolates reached at the exponential phase of growth (OD = 0.5), to obtain reliable sequencing analysis results by extracting a large dosage of DNA. The DNA of isolates were extracted via a BioSpec 3110BX Mini-BeadBeater (Biospec Products, Bartlesville, OK, USA) by mixing 0.1-mm glass beads and Tris-EDTA buffer. The 16s rRNA genes of all isolates were targeted by 1492R or 27F primer [29], and amplified by polymerase chain reaction (PCR) using a thermocycler. The raw sequences of 16 rRNA were identified through the Basic Local Alignment Search Tool (BLAST), which provided the closest relatives from the Gen-Bank database, and MEGA 8 software was used to design a phylogenetic affiliation.

The growth rate of two isolates was determined by measuring the optical density (OD at 600 nm) via a Genesystem 30 Visible Spectrophotometer (Thermo Fisher Scientific, Waltham, MA, USA). The isolates were incubated in tryptic soy broth-urea (TSB-urea) medium with the addition of 3% NaCl and at 15 °C to offer an environment similar to seawater. To observe the ureolysis activity of two isolates, NH_4_^+^ and Ca^2+^ concentration were measured via an ion exchange chromatography (IC). The isolates were incubated in a nutrient broth-urea (NB-urea) medium containing 3% NaCl and 10 mM Ca-lactate at 15 °C by shaking at 200 rpm. To prepare the samples for IC analysis, 5 mL of culture solution was extracted over time, and then supernatants from the extracted samples were obtained by a high-speed refrigerated centrifuge at 4800× *g* for 5 min. The remaining precipitates were recovered for measuring the dry weight of white precipitate and a mineralogical analysis using X-ray diffraction (XRD).

### 2.2. Material and Specimen Preparation

Portland cement (PC) and sand were used as a binder material and fine aggregate, respectively. Bottom ash, obtained from Korea South-East Power Corporation (KOEN, Jinju, South Korea), was added to serve as a bio-carrier to protect the isolated ureolytic bacteria from the harsh environment in the cement matrix. The particle size of bottom ash was 1–4 mm.

**Figure 1:**
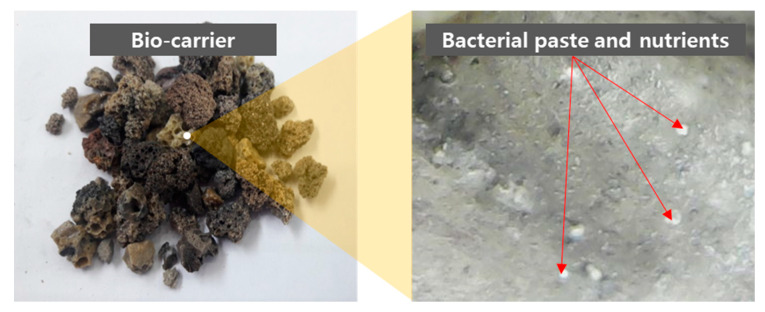
Bio-carriers immobilized with bacterial paste and nutrients at 500× magnification.

The bottom ash was sterilized via an autoclave, prior to the immobilization process. The bio-carrier was immobilized with isolated ureolytic bacterium by immersing the sterile bottom ash in the bacterial culture solution, which contains 3% TSB, 2% urea, and 0.3% bacterial paste, for 10 h. The bottom ash immobilized with ureolytic bacterial culture solution was coated with cement powder, which could prevent leaching of the bacterial paste and substances required for microbial growth during the cement hydration and hardening period. Image of the bio-carriers immobilized with bacterial paste and nutrients is shown in Figure 1.

The water-to-binder ratio was 0.5 and the sand-to-binder ratio was 1.5. The number of isolated bacteria incorporated into the cement matrix in the state of bacterial culture solution and bio-carrier was set to 1.0 × 10^6^ cells/mL, and the bio-carrier-to-binder ratio was 0.4. The mix proportion of the specimens is tabulated in Table 1. The fresh mixtures were cast into a mold 50 × 50 × 50 mm^3^ in size and cured in air for the initial 48 h at room temperature. All specimens were demolded, followed by immersed in water until testing days at room temperature.

### 2.3. Test

In order to analyze the changes in mechanical properties of all specimens, the compressive strength test was performed after 7, 14, 28, 56, and 90 days of curing by using a universal testing machine in accordance with ASTM C109 [30]. The pore size distribution and porosity were measured by means of MIP analysis using an AutoPore IV 9500 (Micromeritics Corp., Norcross, GA, USA) at Korea Basic Science Institute Jeonju Center. The MIP analysis was performed after 7 and 28 days of water curing using fractured specimens having sizes of 3–10 mm. The pressure range used for the MIP analysis was ranged from 1 to 60,000 psia. For XRD analysis of specimens A and C, hydration stoppage by solvent exchange was conducted in accordance with the RILEM TC-238 SCM recommendation [31], after 7 and 28 days of water curing. The XRD analysis was performed via an Empyrean XRD Diffractometer with a line focus 4 kW Cu-Kα X-ray tube (Malvern Panalytical B.V., Almelo, Netherlands) at Smart Open Lab in KBSI. To quantitatively estimate phases in hydrated cement paste, 10.0 wt.% rutile (TiO_2_, Sigma-Aldrich, St. Louis, MO, USA) was added in the specimens as an internal standard material [32,33].

The self-healing efficiency was determined by visual observation and crack width measurement by using a Digibird USB microscope ORT-500 (Digibird, Seoul, South Korea). Single cracks on the faces of the specimens were created by applying pressures through UTM after 28 days of water curing. Before curing for self-healing performance, the initial crack width of all mortar specimens ranged from 0.02 mm to 0.6 mm. The specimens with predefined cracks were cured in three different environments (seawater, tap water, and air) for 28 days, and seawater composition was created using seawater reagent (Sigma Aldrich). Curing in seawater and tap water was carried out according to a wet–dry cycle, which entailed immersing the specimens in seawater at 10 °C or tap water at 25 °C for 2 days, followed by exposing them to the ambient air for 1 day. Following Zhang et al. (2017) [34], the crack-healing ratio *H*_R_ (%) was calculated as follows:

(1) $$H_{R}=\frac{\left(H_{i}-H_{d}\right)}{H_{d}}\times 100$$

where *H*_i_, and *H*_d_ denote initial crack width (mm) and crack width measured after 7, 14, and 28 days of curing (mm), respectively [34]. The mineralogical analysis of precipitates filled in the cracks of E specimens was carried out by XRD analysis.

## 3. Metabolic Characteristics of the Isolated Bacteria

**Figure 2:**
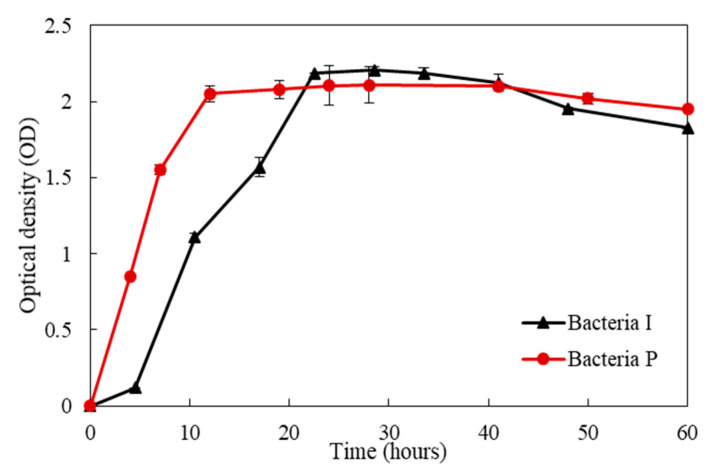
Optical density changes of Bacteria I and P over time.

To understand the basic metabolism of isolated bacteria, the growth rate and ureolytic activity were investigated. Ureolytic bacteria isolated from the West Sea and East Sea were denoted as Bacteria I and P, respectively. Figure 2 displays optical density changes of Bacteria I and P overtime. It should be noted that the bacterial growth curve is represented as lag phase, exponential phase, stationary phase, and death phase; hence, the growth phases of isolated bacteria can be determined by measuring the turbidity of bacterial culture medium which is governed by the number of bacteria [20]. The changes in the turbidity of the culture medium of Bacteria P and I were observed by measuring OD at a wavelength of 600. There was no noticeable lag phase in the growth of Bacteria P, whereas the lag phase of Bacteria I was observed approximately at 5 h (Figure 2). In addition, it took 11 h (OD = 2.1) and 19 h (OD = 2.2) to reach the stationary phase in the growth of Bacteria P and I, respectively, indicating that Bacteria P has a faster growth rate than Bacteria I.

**Figure 3:**
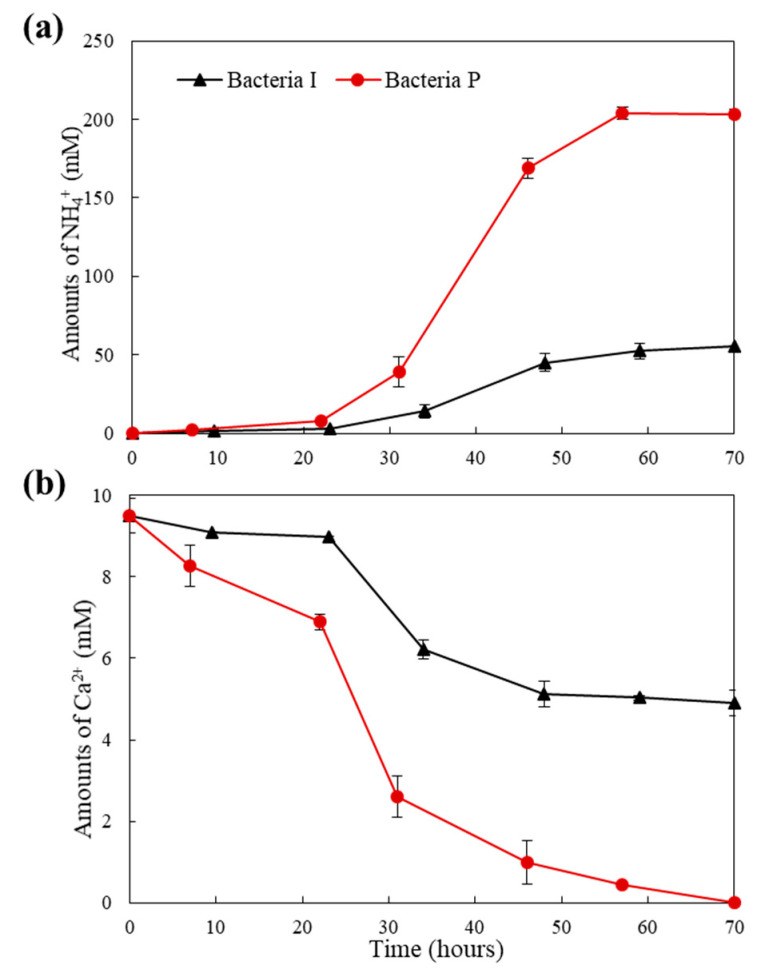
The changes of (**a**) NH_4_^+^ and (**b**) Ca^2+^ concentrations in the culture solution of Bacteria I and P over time.

NH_4_^+^ and CO_3_^2−^ are the main products of ureolysis metabolism, and CaCO_3_ can be formed by combining CO_3_^2−^ and Ca^2+^ released from the cement matrix. It can be inferred that the level of NH_4_^+^ and Ca^2+^ concentration is related with ureolytic activity. The changes of NH_4_^+^ and Ca^2+^ concentration in the culture solution of the two isolates are shown in Figure 3. The NH_4_^+^ final yields of Bacteria P at 70 h was four times higher than that of Bacteria I, and Ca^2+^ was completely consumed, whereas Bacteria I consumed 54% of the initial Ca^2+^ at 70 h. This means that Bacteria P has a higher growth rate and ureolytic performance than Bacteria I.

**Figure 4:**
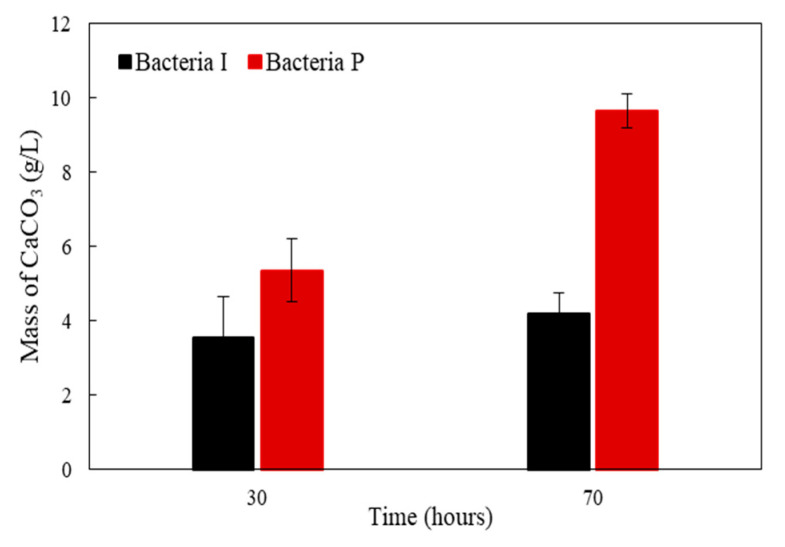
The dry weights of white precipitates formed by Bacteria I and P after 30 and 70 h of incubation.

**Figure 5:**
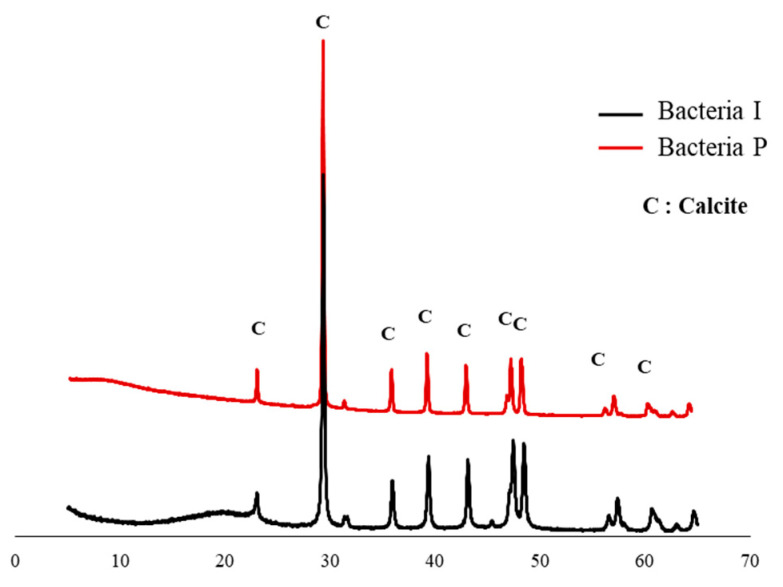
XRD pattern of white precipitates formed by Bacteria I and P.

Figure 4 shows the dry weights of white precipitates formed by Bacteria P and I. The amount of white precipitates formed by the Bacteria P was 1.5 times and 2.3 times higher than those of the Bacteria I at 30 and 70 h, respectively. A noticeable difference in the amount of white crystals by the two isolates was observed, although there was not a significant difference in the OD value, which indicates the amount of nucleation sites for white precipitates (Figure 2 and Figure 4). It is surmised that the ureolysis performance of bacteria could be more important than the nucleation sites. The XRD pattern of white precipitates formed by Bacteria I and P are shown in Figure 5. In general, the crystal phase of CaCO_3_ is divided into aragonite, vaterite, and calcite, according to the bacteria species or various culture environments (i.e., salinity, temperature, pH, etc.) [35,36,37]. The XRD patterns clearly showed that the presence of calcite with a primary indication at 28–30° 2θ (CaCO_3_, PDF# 01-072-1937).

**Figure 6:**
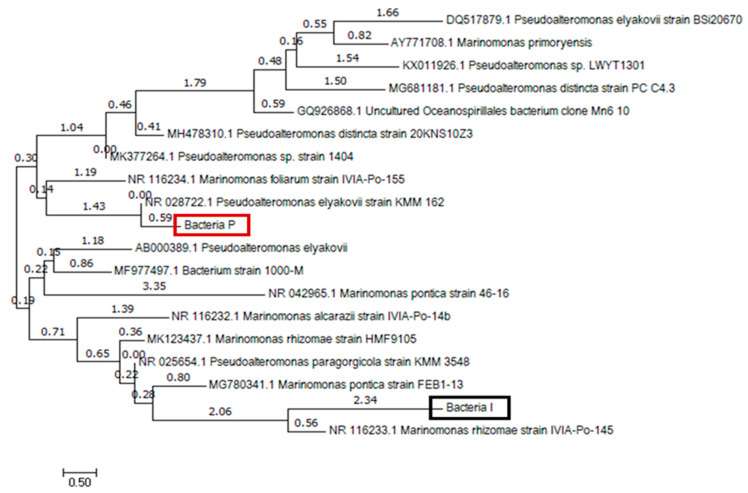
Phylogenetic classification of isolated Bacteria I and P by Maximum Likelihood method.

In order to identify the two isolates, phylogenetic classification of isolated Bacteria I and P by Maximum Likelihood method is shown in Figure 6. The closest species of Bacteria I was *Marinomonas* species, which have a slightly alkaliphilic and high salinity tolerant properties [38]. Bacteria P was most closely related with *Pseudoalteromonas* species. This species is commonly found bacteria in the marine environment and has high metabolic properties, and can survive in nutrient-limited environments [39]. Considering the metabolic capacity of the two isolates, Bacteria P with better performances was used as a self-healing agent in this study.

**Figure 7:**
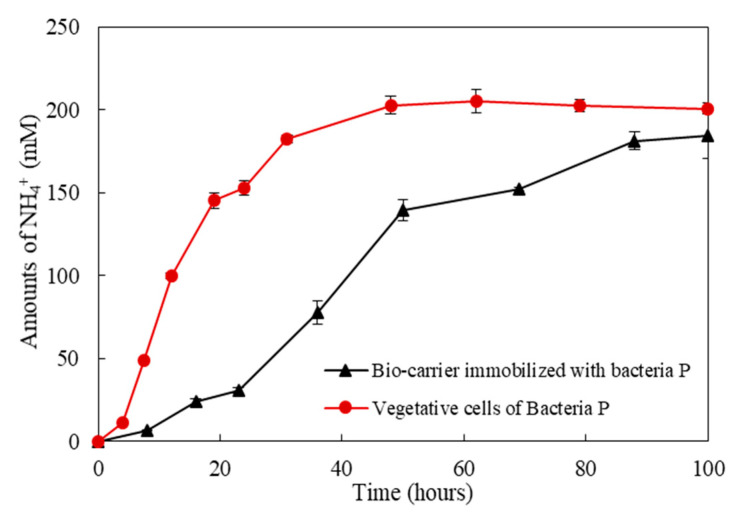
The NH_4_^+^ concentration changes of vegetative cells of Bacteria P and bio-carrier immobilized with Bacteria P in TSB-urea medium over time.

The ureolytic activity of the bio-carrier was investigated by comparing the change in NH_4_^+^ concentration with that of bacterial paste. Figure 7 displays the NH_4_^+^ concentration of vegetative cells of Bacteria P and bio-carrier immobilized with Bacteria P. The maximum concentration of NH_4_^+^ in the culture solution of Bacteria P and bio-carrier was reached after 70 and 100 h of incubation, respectively. The urea degradation rate of Bacteria P vegetative cells was higher than that of the bio-carrier. It is suggested from the earlier findings [40,41,42,43] that the incorporation of a bio-carrier immobilized with bacteria into cementitious materials could improve the microbial viability given that bio-carrier protect the bacteria from harsh environment of cement matrix, whereas it may result in lower ureolysis performance than direct incorporation of vegetative cells.

## 4. Physico-Mechanical Properties and Pore Characteristics of Mortar Specimens with Bio-Carrier

**Figure 8:**
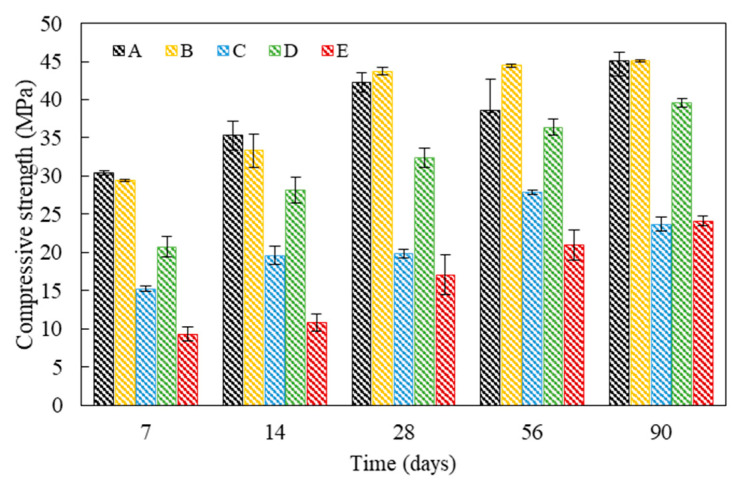
Compressive strength development of mortar specimens after 7, 14, 28, 56, and 90 days of water curing.

**Figure 9:**
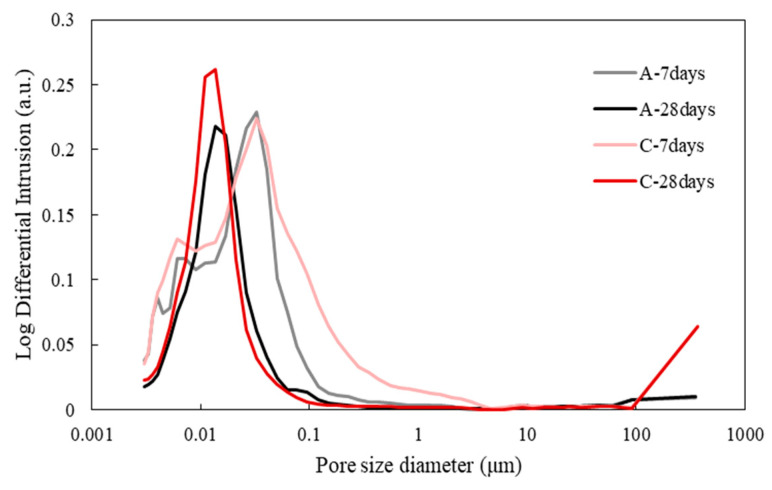
Pore size distribution of specimens without (A) and with (C) Bacteria P after 7 and 28 days of water curing.

The effect of the bio-carrier and bacterial culture solution incorporations on the mechanical properties and pore characteristics of cementitious materials was determined by means of compression test and MIP analysis, respectively. Figure 8 shows the compressive strength development of mortar specimens after 7, 14, 28, 56, and 90 days of water curing. In addition, the effect of newly isolated microbial metabolism on pore characteristics was investigated, since pore distribution and strength development could be influenced by bacteria types and bacterial metabolic capacity [43,44,45]. The pore size distribution of specimens A and C are shown in Figure 9 and their pore characteristics are tabulated in Table 2.

The strength of specimen C directly incorporating the Bacteria P culture solution was 52.9% lower than that of specimen A at 28 days (Figure 8). On the other hand, the average pore diameter and porosity of specimen C were 1.53 mm and 2.59% lower than those of specimen A, respectively, at 28 days (Figure 9 and Table 3). It can be inferred that the direct incorporation of bacterial culture solution could have a negative effect on strength development due to a decrease in hydration kinetics, yet the pore size could be decreased by bacterial CaCO_3_ precipitation metabolism. In addition, although CaCO_3_ formed by bacteria filled the internal voids or cracks, it had no significant effect on strength, signifying that the compressive strength development of the specimens was mostly governed by the degree of hydration. On the other hand, the strength of specimen D incorporating the bio-carriers was 1.6 times higher than that of specimen C after 28 days (Figure 8). Possible elucidation lies that the usage of bio-carrier as a self-healing agent could alleviate the retardation of cement hydration, while the direct addition of bacterial culture solution to cement inhibited the contact of cement clinkers with water, thereby decreasing the compressive strength.

Meanwhile, the strength values of specimens A and B were not significantly different, indicating that the incorporation of bottom ash may not have a noticeable influence on the strength (Figure 8). The strength values of specimen C barely showed an increase of strength values, while the strength of specimens D and E slightly increased until 90 days of curing (Figure 8). It is suggested that the incorporation of the bio-carrier could have a positive effect on the sustainable metabolic activity of bacteria in concrete, since the numerous pores of bottom ash could store bacteria, nutrients, and moisture. In addition, the moisture stored in the pores could promote the further hydration [46], thereby possibly enhancing the long-term hydration degree. Nevertheless, the strength of specimen E was 42.12% lower than that of specimen D after 90 days of water curing and, as a result, specimen E showed the lowest strength values at all ages (Figure 8). In terms of long-term durability, the incorporation of bio-carriers instead of a bacterial culture solution could be a more suitable method to achieve the desirable strength development.

**Figure 10:**
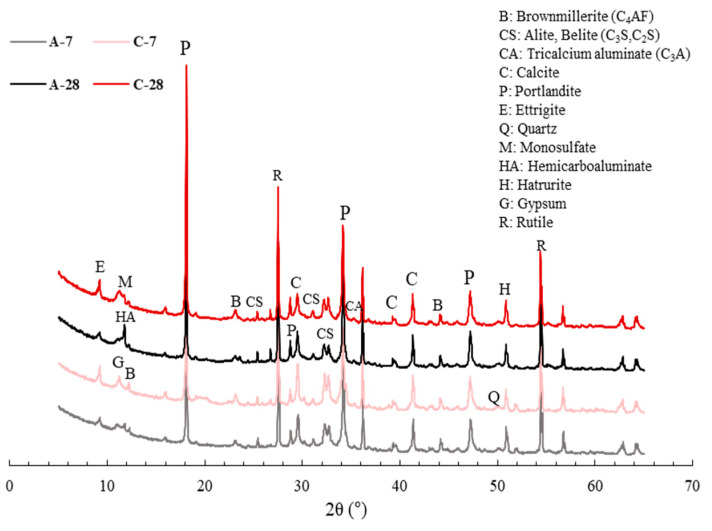
XRD patterns of paste specimens with and without Bacteria P after 7 and 28 days of water curing.

Figure 10 shows the XRD patterns of specimens A and C after 7 and 28 days of water curing. As reported in earlier works [47,48], the use of bottom ash as an aggregate does not have a distinguishable effect on the evolution of hydrates. It should, therefore, be mentioned that the phase identification of the A and C specimens was carried out in order to observe the effect of bacterial culture solution on hydration products. As mentioned in several previous studies [49,50], there were a few differences in the hydrate phase. However, there were quantitative differences in the clinker phase and minerals in cement (C_3_S, β-C_2_S, C3A, C_4_AF, lime, periclase, gypsum, anhydrite), and hydration products (portlandite, calcium-silicate-hydrate (C-S-H) gel, ettringite), owing to hydration delay by the incorporation of bacteria and nutrients. Table 3 displays the quantitative change of the phases in specimens A and C via Rietveld refinement. Specimen A had less clinker and more hydrates than specimen C, especially for the amorphous content which refers to the presence of C-S-H gel, indicating that the degree of hydration of specimen A was higher than specimen C. On the other hand, more calcite was observed in specimen C incorporating the bacterial culture solution, although calcite could be generally formed by a natural carbonation or autogenous healing system [51,52]. It can be said that Bacteria P could survive and perform the CaCO_3_ precipitation metabolism in the harsh environment of concrete. In addition, the carbonation process may be limited, since it is difficult for CO_2_ required for the carbonation process to enter the specimens by water curing.

## 5. Self-Healing Performance of Mortar Specimens with Bio-Carrier

**Figure 11:**
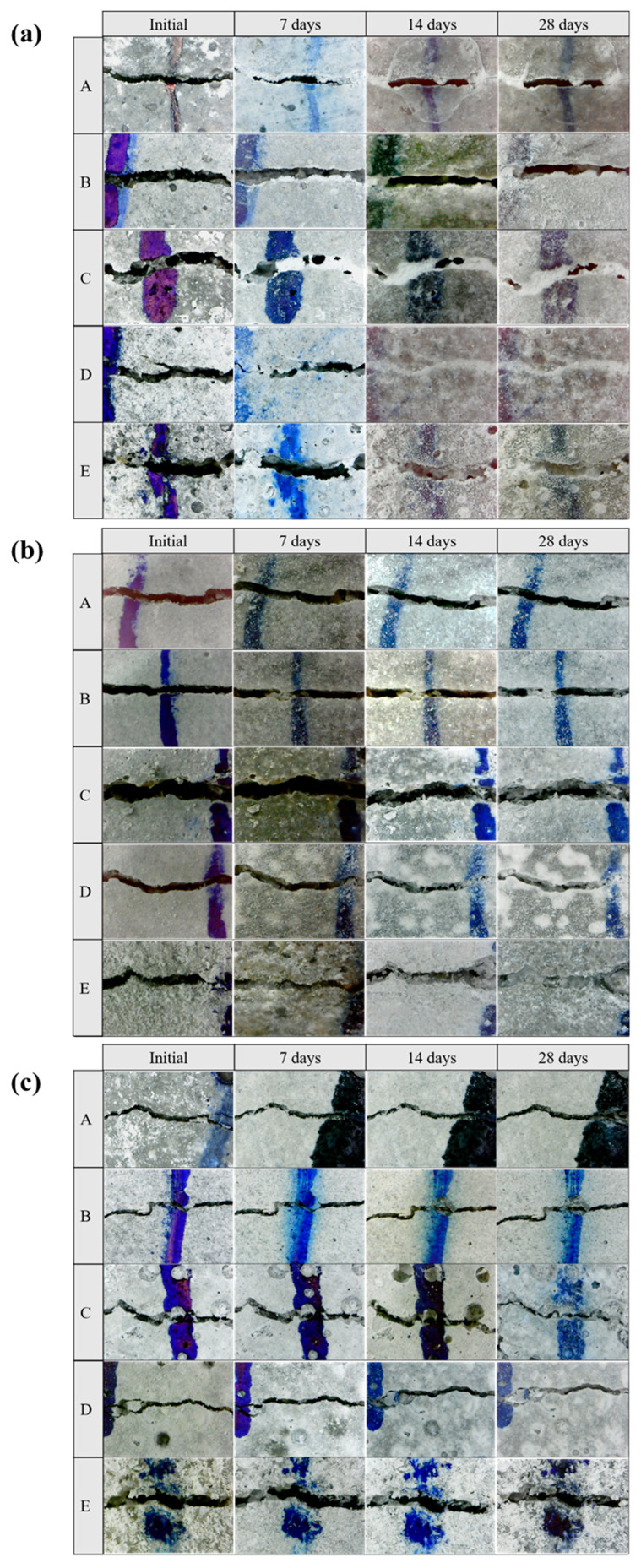
Microscopic observation of crack healing of five types specimens treated in (**a**) seawater, (**b**) tap water, and (**c**) air environment after 7, 14, and 28 days at 500× magnification.

**Figure 12:**
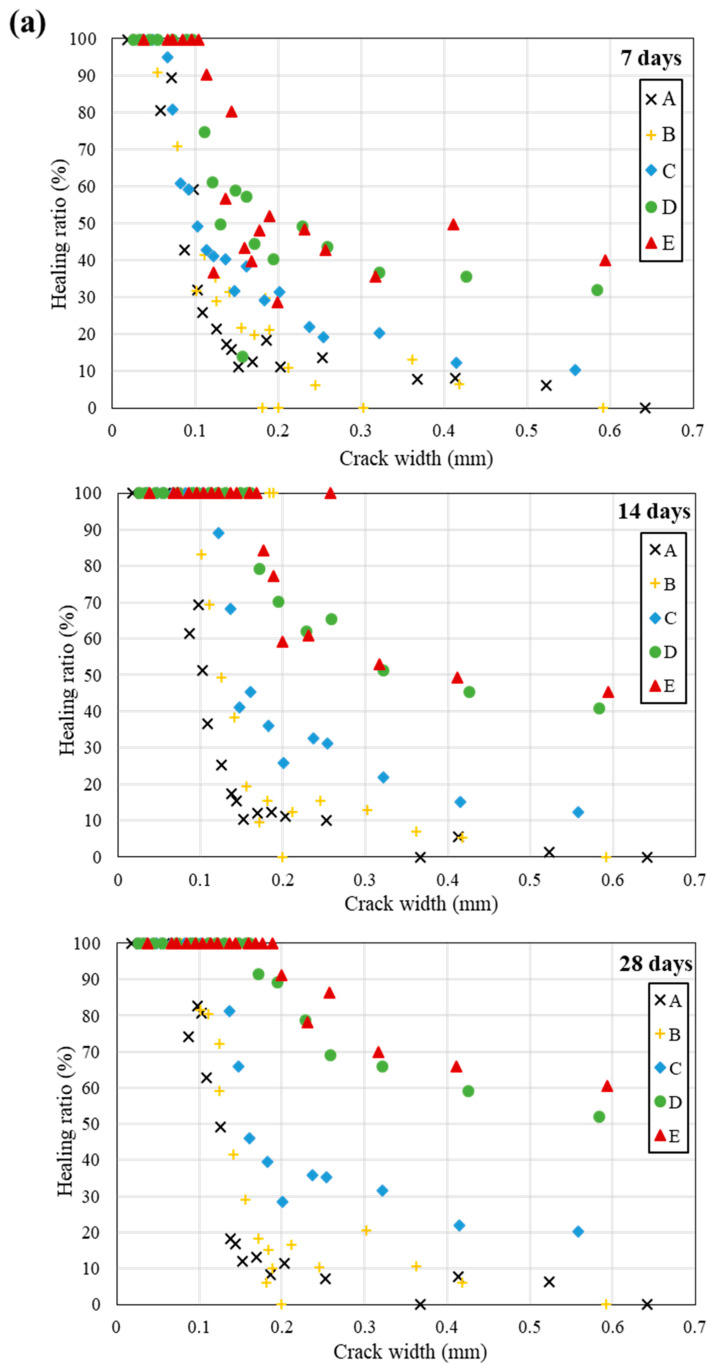

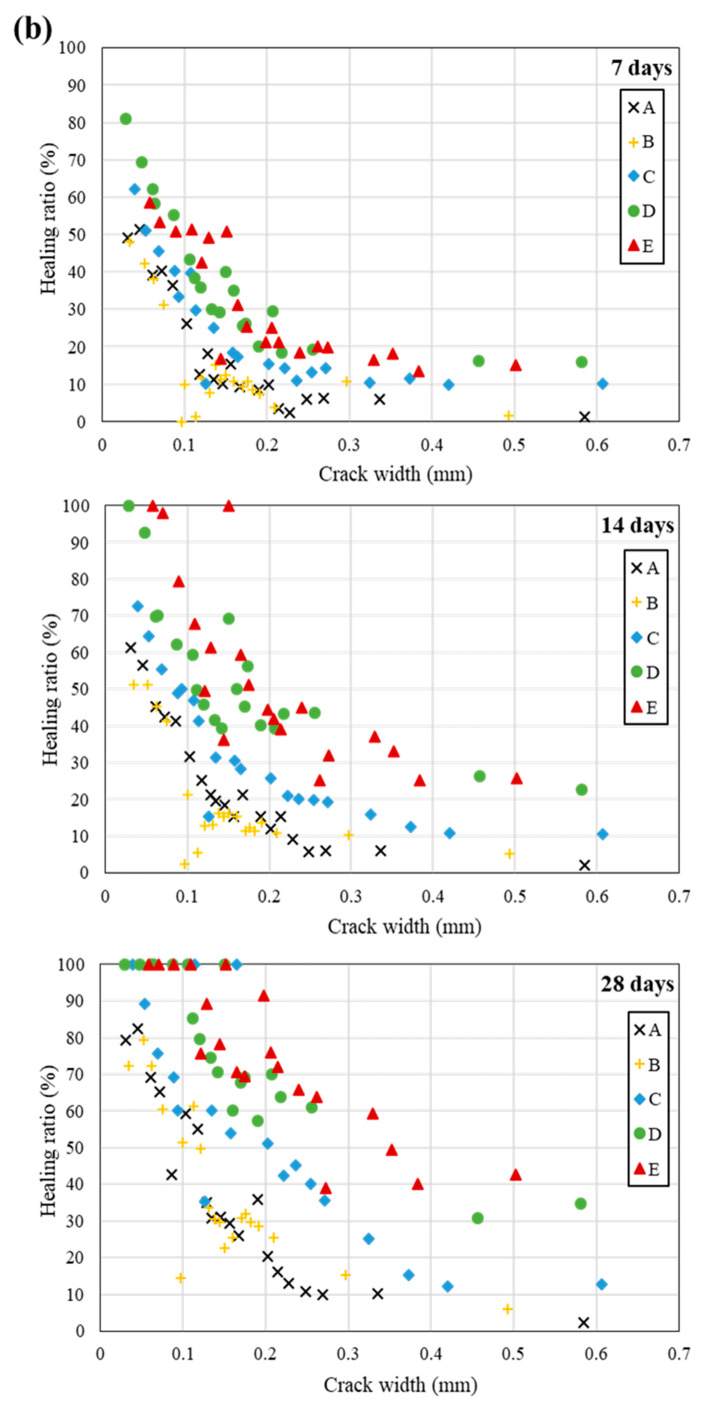

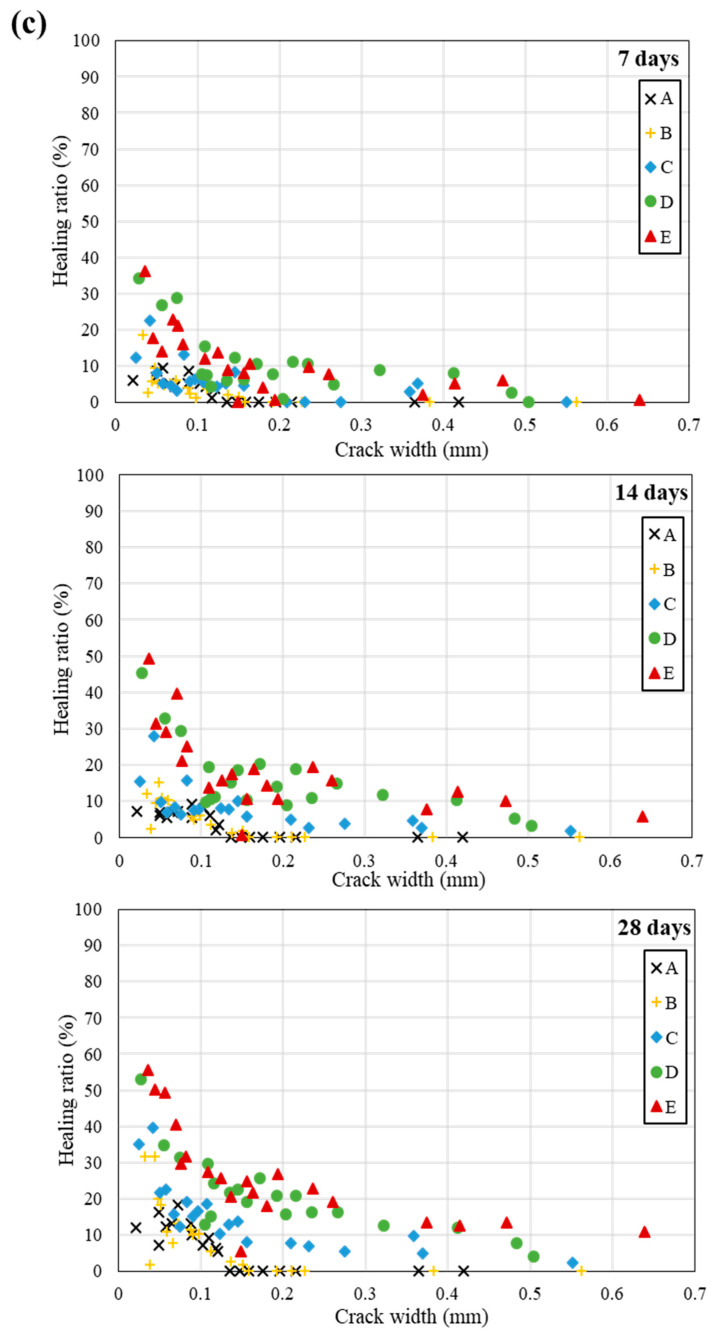
Healing ratio values of five types specimens treated in (**a**) seawater, (**b**) tap water, and (**c**) air environment after 7, 14, and 28 days.

**Figure 13:**
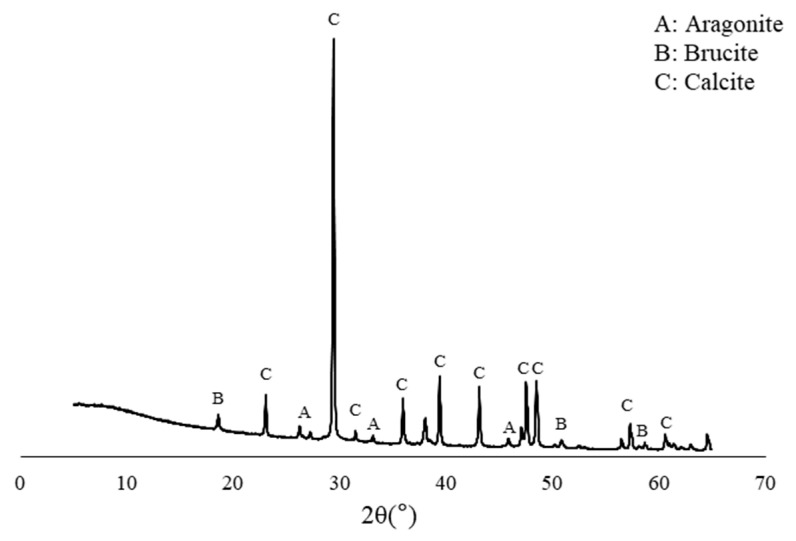
XRD pattern of white precipitates in crack zone of E specimens treated in seawater for 28 days.

Considering various environments around real concrete structures, the self-healing efficiency of all specimens was investigated in three environments (seawater, tap water, and air). In addition, the wet–dry cycle was used to supply bacteria with adequate air and moisture required for active metabolism. The microscopic observation of crack healing of five types specimens treated in three environments are shown in Figure 11. In addition, Figure 12 shows the healing ratio values of five types specimens treated in three environments. The average value of the widths measured over time by randomly selecting 20 widths in initial crack width from 0.02 to 0.6 mm of 5 types specimens (A, B, C, D, and E) with predefined cracks. Moreover, XRD pattern of white precipitates in crack zone of specimen E treated in seawater for 28 days is shown in Figure 13.

The cracks were filled with crystals formed by the bio-mineralization of bacteria or by the autogenous healing system of cementitious materials itself (Figure 11). The specimens C, D, and E, which contained bacterial culture solution or bio-carrier, showed a remarkable healing performance regardless of the applied environments (Figure 12). It can be assumed that the bio-mineralization has better self-healing performance than the autogenous healing system of cementitious materials. Nevertheless, the healing ratio by microbial metabolism was relatively lower when treated in an air environment than in seawater or tap water environment (Figure 12). It can be seen that proper moisture with air play an important role in the bio-mineralization metabolism of bacteria [20]. The highest healing ratio was observed when the specimens were treated in seawater, and only specimens treated in seawater had a completely healed width (A = 0.03 mm, B = 0.032 mm, C = 0.055 mm, D = 0.096 mm, and E = 0.104 mm) after 7 days (Figure 12a). It is indicated that seawater, in which various metal ions and nutrients are present, could have a positive effect on the survival and metabolic activity of bacteria. In addition, as shown in Figure 13, the white precipitates of specimen E were composed predominantly of calcite, brucite, and aragonite, whereas the precipitate formed by Bacteria P was mainly calcite, as shown in Figure 5. It is suggested that an autogenous healing system could accelerate the closure of cracks by combining Ca^2+^ and Mg^2+^ remaining in the cement matrix with CO_3_^2−^ and SO_4_^2−^ present in seawater.

Meanwhile, the healing ratios of specimens D and E were not significantly different, although specimen E incorporated both the bacterial culture solution and the bio-carrier (Figure 12). It can be inferred that direct incorporation of bacteria into cementitious materials may cause not only a reduction of microbial viability in extreme concrete environments, but also impose limitations of sustainable metabolism due to a loss of nutrients during the mixing process. On the other hand, the healing ratio of specimen D was higher than that of specimen C in the three different environments (Figure 12). It is suggested that the bio-carrier could offer space for survival and CaCO_3_ precipitation metabolism. In addition, the bio-carrier coated with cement powder could overcome the limitation of nutrient loss during the mixing process [34,53], thereby improving the self-healing efficiency and microbial viability. The healing ratios of the specimens treated in air were the lowest; nevertheless, specimens D and E incorporating the bio-carrier had a higher healing ratio than that of others treated in air (Figure 12b,c). It can be inferred that bottom ash has numerous pores, which can provide micro-environments for bacteria and storage space for nutrients or moisture, thereby improving the microbial viability and sustainable self-healing efficiency.

Many researchers have introduced bio-carriers to improve the microbial viability in the harsh environment of concrete and to maintain the self-healing performance. In this paper, bottom ash immobilized with Bacteria P was used as a bio-carrier. In addition, the results of healing ratio measurement indicate that the bio-carrier could be used as a self-healing agent for long-term durability and sustainable self-healing performance of concrete structures. However, determining the self-healing efficiency by measuring the healing ratio through only microscopic observation could have a certain limitation, since only the width of the surface cracks overtime is measured. In general, crack healing performance could be influenced not only by crack width but also by depth. Indeed, the surface cracks of the specimens treated in seawater were quickly healed, while the internal cracks were not completely filled, since closure of the surface cracks made it difficult for moisture and air to enter the interior. Therefore, further studies to better understand the healing ratio according to the crack depth, such as non-destructive testing and detection of cracks using an ultra-sonic technique, should be carried out with the aim of evaluating the potential of prolonged durability of concrete structures.

## 6. Concluding Remarks

The effects of incorporating a bio-carrier immobilized with ureolytic bacteria isolated in a marine environment on the self-healing efficiency and mechanical properties of a cementitious material were evaluated in this study. The obtained results showed that the incorporation of the bio-carrier into cementitious materials had a positive effect on the strength and crack-healing performance, compared to the direct incorporation of vegetative cells. The major findings of this study can be summarized as follows:

(1) The rate of urea degradation in vegetative cells was higher than that of bacteria immobilized in carriers. This finding indicates that the bio-carrier could retard the metabolic activity, yet it could protect the bacteria from the extreme environment of the concrete.
(2) The highest healing ratio was observed in the specimen incorporating the bio-carrier. It is suggested that the bio-carrier not only created a space for bacteria in concrete, but also stored moisture and nutrients, thereby improving the long-term self-healing efficiency.
(3) The specimens incorporating bacteria and the bio-carrier had a higher healing ratio when treated in seawater than those treated in tap water or in air environment. It can be said that the various ions present in the seawater have a positive effect on promoting the metabolism of bacteria isolated in the seawater.
(4) The crack healing ratio of mortar specimens was determined by measuring the surface crack width through microscopic observation in this paper, although self-healing efficiency could influenced not only by crack width but also by depth. Further studies on the healing ratio of crack depths are needed to secure long-term durability.

## Figures and Tables

**Table 1:** Mix proportion of mortar specimens with and without bio-carrier by mass ratio.

Specimen ID	Cement	Water	Bacterial Solution *	Sand	Bottom Ash	Bio-Carrier
A	1.0	0.5		1.5		
B	1.0	0.5		1.5	0.2	
C	1.0		0.5	1.5		
D	1.0	0.5		1.5		0.2
E	1.0		0.5	1.5		0.2

* Bacterial solution is cultural medium of Bacteria P incubated in 15 °C for 10 h. The solution is consisted of 3% TSB, 2% urea, and 0.3% bacterial paste.

**Table 2:** Pore characteristics of specimens without (A) and with (C) Bacteria P.

	A		C	
	7 Days	28 Days	7 Days	28 Days
Average pore diameter	14.50 nm	13.24 nm	16.77 nm	11.71 nm
Median pore diameter *	24.19 nm	15.33 nm	30.91 nm	13.08 nm
Porosity	32.16%	27.09%	38.60%	24.50%

* Median pore diameter at 23,508.67 psia and 27.645 m^2^/g.

**Table 3:** Quantitative X-ray diffraction analysis by Rietveld refinement (g/100 g of binder).

Phase Name	A		C	
	7 Days	28 Days	7 Days	28 Days
C_3_S	3.2	1.2	7.8	2.1
β-C_2_S	8.9	5.9	9.3	8.6
C_3_A	0	0	2.3	0
C_4_AF	2.6	0.1	4.9	2.1
Quartz	1.1	2.8	2.2	3.1
Portlandite	15.4	16.1	14.5	17.3
Calcite	5.8	6.1	6.5	8.3
Gypsum	1.4	0.8	2.5	0.4
Ettringite	3.9	3.4	7.7	6.2
Anhydrite	0.1	0	0.6	0.5
Lime	3.3	0.8	4.8	0.6
Amorphous	54.3	62.8	36.9	50.8
